# Online Methods for Locating Medical Marijuana Dispensaries: Practical Considerations for Future Research

**DOI:** 10.26828/cannabis.2018.02.003

**Published:** 2018-07-07

**Authors:** Eric R. Pedersen, Megan Zander-Cotugno, Regina A. Shih, Joan S. Tucker, Michael S. Dunbar, Elizabeth J. D’Amico

**Affiliations:** 1 RAND Corporation, Santa Monica, CA, USA; 2 RAND Corporation, Arlington, VA, USA; 3 RAND Corporation, Pittsburgh, PA, USA

**Keywords:** marijuana, medicinal cannabis, dispensaries, neighborhood

## Abstract

Knowing the names, locations, and signage of medical marijuana dispensaries is critical for assessing how marijuana availability affects neighborhood quality and marijuana use, yet no detailed methods for locating and coding dispensaries are published. Limitations regarding accuracy of official records, unregulated businesses, and the size of areas where dispensaries are located make it difficult to accurately capture all open dispensaries in any area. In this study, we test a practical and feasible method to collect a point-in-time portrayal of medical marijuana dispensaries in a large urban area lacking an official record of these businesses. Using publicly-available Internet sources alone, we collected the name, address, phone number, signage, and open/closed status of medical marijuana dispensaries in Los Angeles County between October 2016 and February 2017. Data were verified by calling a subset of dispensaries and comparing data against a list of licensed dispensaries. Our methods yielded 872 unique dispensaries in Los Angeles County, of which 470 were open. Most open stores were discernable by a green cross sign; however, few had names that clearly indicated the store sold marijuana. Data verification procedures showed that Internet sources were able to locate nearly all dispensaries in the county that were then verified with non-Internet methods, such as calling the businesses to confirm information. This study is significant as it provides methodology that can be replicated in other metropolitan areas, facilitating comparisons across databases in different locations and regulatory environments. However, caution should be taken when solely using Internet sources. Accurate information on dispensary names, locations, and signage can advance research and provide important information for policy decisions. Methods for enhancing the online methods described in this study are discussed.

In the 22 years since marijuana for medical purposes was legalized in California, medical marijuana dispensaries have proliferated throughout the state. Though the actual size of the medical marijuana market in California is not known, some industry sources estimate that it is a billion dollar industry. In November 2016, California voters passed Proposition 64, the Adult Use of Marijuana Act, making it legal for individuals aged 21 years or older to grow and sell marijuana for recreational use. The market will no doubt continue to flourish, as licenses for recreational stores became available in January 2018, expanding access to marijuana to all California residents aged 21 and older regardless of medical conditions.

One primary argument in opposition to marijuana legalization was the unknown effects of how availability of the drug would affect youth and adult use and normative behaviors. Several studies in the United States on alcohol retail stores have shown that access to alcohol outlets, measured by either density or proximity to retail stores, is associated with drinking behaviors (Gmel, Holmes, & Studer, 2016; Shih et al., 2015). To date, however, there are very few studies addressing how proximity to and density of marijuana dispensaries may affect adolescent and adult marijuana use (Freisthler & Gruenewald, 2014; Shi, Meseck, & Jankowska, 2016); how dispensary locations are related to other demographic, economic, and social environmental factors, such as crimes and physical violence, within surrounding neighborhood(s) (Freisthler, Gruenewald, & Wolf, 2015; Freisthler, Ponicki, Gaidus, & Gruenewald, 2016); and whether dispensaries are disproportionately located in areas that target low-income or minority populations (Thomas & Freisthler, 2016). These and future studies can help to answer research questions that are essential to informing policies as states, counties, and cities develop guidelines regarding locations of dispensaries.

The first step to advance this important research is to locate medical marijuana dispensaries to document visibility of dispensaries and develop metrics of their density and proximity in neighborhoods. Though researchers have attempted to locate medical marijuana dispensaries in California, Colorado, and Washington (Freisthler & Gruenewald, 2014; Freisthler et al., 2016; Hunt, Pacula, & Jacobson, 2014; Kepple & Freisthler, 2012; Kilmer et al., 2013; Nunberg, Kilmer, Pacula, & Burgdorf, 2011; Pacula, Chriqui, Reichman, & Terry-McElrath, 2002; Saloga, Boustead, Jacobson, Pacula, & Anderson, 2013; Shi, 2016; Thomas & Freisthler, 2016), there are no detailed methods published. It is crucial to develop standardized, comprehensive, and practical methods to locate medical marijuana dispensaries to better understand the effects of marijuana availability on neighborhood quality and marijuana use. This can pave the way for future methodological work on locating and coding outlets that sell marijuana for recreational purposes.

## The Difficulty with Locating Dispensaries in Los Angeles County

Los Angeles County represents an area where it is particularly difficult to locate and count marijuana dispensaries given that it is a very large area and there are no city level records as of the time of this writing. Thus, the current study focuses on whether online methods can be used to collect location and other information about dispensaries in the Los Angeles area. This is an important undertaking given that many other large metropolitan areas across the United States may soon face similar challenges. Due to fluctuations in store closings and new store openings, differing medical marijuana dispensary regulations across cities and neighborhoods within Los Angeles County, and operation of stores without business licenses, it is not possible to utilize official records documenting the open dispensaries in the county, as no such comprehensive list exists (Freisthler, Kepple, Sims, & Martin, 2013). The California Department of Public Health’s Office of Medical Cannabis Safety is currently tasked with developing statewide standards and regulations for medical marijuana dispensaries, and the office began issuing new business licenses to dispensaries for both medical and recreational use in January 2018.

The broader Los Angeles County expands over 4,750 square miles and contains 88 incorporated cities such as Long Beach, Glendale, Santa Clarita, Torrance, Pasadena, and Inglewood. Some estimates on the Internet report there are over 1,700 open marijuana dispensaries in the city of Los Angeles alone, which represents the largest city in Los Angeles County. However, official records at the city level are non-existent at the time of this writing. For example, business tax records do not fully capture the extent of dispensaries. In 2016, the Los Angeles city controller found that 756 dispensaries in the city of Los Angeles held Business Tax Registration Certificates (BTRC), but just 139 dispensaries obtained BTRCs in 2017 that were determined to be in compliance with the city’s tax regulations (Los Angeles Controller, 2017). Due to stores not being legally compliant, there is constant fluctuation in stores’ open and closed status across the city. The Los Angeles City Controller states that the Los Angeles City Attorney’s Office has filed criminal charges against 563 dispensaries and the city attorney’s website claims to have “shut down more than 800 of the unlawful dispensaries throughout the city” since 2013 (Los Angeles City Attorney, 2017). As stores close or are shut down by the city, more stores emerge in their place and the closed stores may simply relocate or change names.

The issue of fluctuation in dispensaries and lack of official records is likely to plague other cities as they develop regulations around medical marijuana dispensaries, and for some, recreational marijuana dispensaries. Similarly, any study that uses lists of licensed dispensaries alone will no doubt miss many dispensaries that are unlicensed and unregulated. For example, Shi and colleagues (Shi et al., 2016) used the directories of licensed marijuana stores from the Enforcement Division of Colorado Department of Revenue to locate marijuana dispensaries in Colorado, but given the lenient laws around cultivation in the state, individuals and co-operatives have found ways to grow and sell the drug in an unregulated “grey market” (Colorado Office of the Governor, 2016). Outside of marijuana dispensaries, official lists of licensed stores have not adequately captured all the retail shops selling Electronic Nicotine Delivery Systems (ENDS), which are e-cigarettes and related products (typically sold in combination with nicotine “e-liquids”) that deliver nicotine to an individual in aerosolized form. Such specialty “vape shops” and other outlets selling ENDS (e.g., convenience stores) have proliferated in recent years, coinciding with the dramatic rise in popularity of these products (United States Department of Health and Human Services, 2016). For example, researchers in Florida reported that only 32.5% of the stores they verified as selling ENDS were actually in the list of licensed tobacco retailers from the Florida Department of Business and Professional Regulation (Kim et al., 2016).

## The Limitations of Prior Research on Locating Medical Marijuana Dispensaries

Recent studies that have focused on how to locate medical marijuana dispensaries have limitations that need to be addressed. First, descriptions of methods for locating dispensaries are typically limited to a few sentences, making it difficult to replicate methods. For example, researchers describe using websites designed to search a particular area for medical marijuana dispensaries (e.g., WeedMaps, www.weedmaps.com) and Internet search engines (e.g., Yelp; www.yelp.com) to locate marijuana dispensaries, followed by in some cases, very extensive efforts such as calling, driving by, or visiting stores to ensure they are open (Freisthler & Gruenewald, 2014; Freisthler et al., 2015; Freisthler et al., 2016; Lipperman-Kreda, Juliet, Morrison, & Bridget, 2014). However, there is not enough detail in these brief methods sections to replicate findings for one’s own research purposes. In addition, the methods that have been used have not been examined in the context of the other limitations we describe below and further efforts to verify that these methods are adequate are necessary.

Second, prior work does not make mention that any data collection period represents a point-in-time snapshot, nor is there mention that a plan is needed for updates to any dispensary database. As with any type of business, fluctuations in store closings and new store openings make continual updating of any medical marijuana dispensary database necessary, as data collected at any one time point would be an accurate reflection of the number and density of stores *for only a brief period of time*. For example, researchers collected location information for marijuana dispensaries in Long Beach, California beginning in January 2012 (Freisthler et al., 2016). At the start of the study, they identified 37 open dispensaries, but two years later in December 2013, only five of these dispensaries were identified as open. Given the turnover, it is necessary for researchers to develop methods that can feasibly be replicated for continuous updating of location data, particularly if researchers are attempting to determine if proximity to and density of marijuana dispensaries is associated with marijuana use among the public. For example, if the goal is to examine how dispensary proximity and density are contemporaneously associated with neighborhood characteristics (e.g., poverty levels in a neighborhood, use of marijuana among neighborhood residents), using outdated dispensary data will result in erroneous conclusions. Thus, methods will need to be practical and efficient in order to be continuously updated in an effort to be as accurate as possible.

Third, some of the more extensive data collection efforts, such as driving and/or calling every dispensary were feasible in previous work because the search was constrained to a smaller area; such as in Long Beach, California (where only 37 open dispensaries were located) (Freisthler et al., 2016) and Sacramento, California (where only 16 open dispensaries were found) (Lipperman-Kreda et al., 2014). In areas that are much larger, such as Los Angeles County, it is not feasible due to budget constraints and the constant fluctuation of these businesses to verify the location data of medical marijuana dispensaries through these more extensive means. It is more practical to use publicly available online information; however, research is needed to better understand how online websites can be used to adequately capture dispensary locations. Detailed methods for how to replicate these methods are also needed, so that researchers can find ways to improve upon the methods and use them for their own purposes.

Finally, no study to date has described signage or store names, presumably because this information has not been collected. Signage, such as a green cross or a marijuana leaf on exterior doors or windows, is especially important with respect to public awareness and perceived accessibility. For example, stemming from work examining storefront advertising of cigarettes, alcohol, and fast food (Celebucki & Diskin, 2002; Hillier et al., 2009), it likely makes a difference whether an individual lives near a dispensary designated by a marijuana leaf on the door versus a dispensary with no sign to indicate that the store sells marijuana. Lack of signage may still influence exposure to marijuana and normative beliefs, but without data on signage, the pathways through which outlets may influence adolescent and adult marijuana use are unclear. Similarly, if a store has a name that is clearly related to marijuana (e.g., Doctor 420’s Pot Shop), someone may easily ascertain that marijuana is sold inside, versus a store with a more ambiguous name (e.g., Beach City Patient Collective). Information on signage and store names could help policymakers make decisions about effects of signage and store names on increased marijuana use or degradation in neighborhood quality. Note that names of stores throughout this paper were fabricated for illustrative purposes and do not intend to represent actual businesses.

This paper addresses many of the limitations of previous work by detailing a specific plan for locating dispensaries in large metropolitan areas using online methods and specific lower cost verification procedures. We utilized this plan within Los Angeles County, an area that has a large population (over 10 million in 2017) living in a large area, but with a dense distribution of medical marijuana dispensaries located within clearly identified boundaries. We describe detailed methods to create a comprehensive database that can be updated regularly. This database can be an invaluable resource to researchers looking to explore changes in medical marijuana and/or recreational outlet number and density over time. This latter point is especially relevant due to recent legalization of recreational marijuana sales in California and other states. Because we utilize online website sources alone, it is anticipated that methods developed in this study for Los Angeles could be easily replicated in other metropolitan areas (e.g., San Francisco, Seattle), facilitating comparisons across databases in different locations and regulatory environments.

## METHOD

### Databases and Search Methods

Data were collected from October 2016 through February 2017. We searched for and located medical marijuana dispensaries in Los Angeles County based on all 526 county zip codes. To ensure the feasibility of continually updating this database, our methods were based solely on publicly available online information collected by a single data extractor. Data verification procedures were conducted by an additional two coders.

Given that no publicly available official database existed, we collected dispensary data from five websites: StickyGuide (www.stickyguide.com), WeedMaps (www.weedmaps.com), Yelp (www.yelp.com), Leafly (www.leafly.com), and Where’s Weed (www.wheresweed.com). Similar to other public websites with business information (e.g., Google, Yellow Pages), these websites offer searchable databases of dispensaries by name and address, as well as customer reviews of the stores, phone numbers, and store hours. Researchers have used websites such as these to help locate address data for medical marijuana dispensaries across a number of cities in California, including Long Beach and Sacramento (Freisthler & Gruenewald, 2014; Freisthler et al., 2016; Kepple & Freisthler, 2012; Thomas & Freisthler, 2016). In the current study, for the marijuana websites, we located stores by entering each of the Los Angeles County zip codes into the website’s search bar. For Yelp, which contains businesses beyond marijuana dispensaries unlike the other websites, we entered multiple search terms (e.g., “marijuana dispensaries,” “cannabis clinics”) into the “find” search bar and each of the zip codes into the “near” search bar.

We first collected location information regarding medical marijuana outlet business sources from the two online marijuana databases (StickyGuide and WeedMaps) that appeared most often in the prior literature, and were recommended by researchers who had used these websites to locate medical marijuana outlets in prior work. Yelp was also initially identified from the prior research and from consultation with researchers as a resource that should be used in coordination with the marijuana websites. To assess whether any dispensaries in Los Angeles County that had an online presence were not identified by these three websites, we then searched two more marijuana websites (Leafly and Where’s Weed) for all zip codes within three large neighborhoods chosen at random that covered 28 zip codes (Venice Beach, West Hollywood, and Long Beach). This allowed us to see if either of these two websites located additional dispensaries beyond those that were found using StickyGuide, WeedMaps, and Yelp. With these preliminary searchers, we began seeing that Leafly and Where’s Weed identified dispensaries not found in the original three sources; thus, we then searched these two additional online sources for dispensaries in all 526 zip codes to ensure that we obtained every possible dispensary via these online methods.

We originally planned to extract data from Medical Marijuana Locators (www.medicalmarijuanalocators.com); however one month into data collection, we reached a “404 error” page when attempting to visit this website, indicating that this website was no longer available. Thus, we selected “Where’s Weed” as the fifth source. We excluded dispensaries outside of Los Angeles County as well as dispensaries that did not have a physical location (i.e., those that were indicated as delivery only). As such, we did not collect data from dispensaries that were clearly labeled as delivery-only dispensaries (i.e., no brick-and-mortar store front). The findings for the delivery-only stores presented below represent dispensaries that were determined to offer delivery after we had completed initial data collection that led us to believe the dispensary had a physical location where customers could purchase marijuana. Of note, many of the dispensaries with store fronts and physical addresses offered delivery, but we did not collect and document this information.

### Data Extraction

We extracted the following data from each website: name of store, address (street, city, zip code), phone number, date store was opened, and current open or closed status. We documented the website(s) on which each dispensary was listed and made note of inconsistent or duplicate information. Open/closed status was determined through a variety of methods such as by viewing the latest update to the dispensary menu or the date of the most recent comment on the websites, posted hours or “closed” indicator on Yelp and Google, searching for the store name in Google and noting if the store was closed as indicated in news articles after large-scale police closures of stores, and in some cases, reviewing the social media pages for the stores and noting the most recent activity. When open/closed status could not clearly be resolved, the dispensary was indicated as having “unknown” status.

We also coded whether the store name clearly indicated that the store sold marijuana using three codes: (1) clear indication (e.g., The Pot Joint), (2) no indication (e.g., Heavenly Remedies Collective), and (3) possible indication (e.g., It’s Easy Bein’ Green). For all dispensaries, we located signage by reviewing user- and store owner-posted pictures of the store front and using Google Maps to view the store front, with particular attention paid to (1) a green cross, (2) a marijuana leaf or paraphernalia picture, and (3) the word “marijuana” or a clear variant of it (e.g., “weed” or “pot”). For this latter signage indicator, the word(s) could have been part of the store’s name as long as there was a sign outside with the word(s) on it. In addition, we noted (4) if stores indicated if they were “Prop D compliant” or “Pre-interim control ordinance (ICO).” Although this type of signage was not a requirement, the label ostensibly indicates the store had been open since before a city moratorium on new dispensaries went into effect in 2007 (see below for description). We coded signs if they appeared on exterior doors, windows, walls, or anything visible from the street as one was walking by or driving by. Though most dispensaries have tinted or covered windows, we did not include any devices (e.g., bongs, vaporizers) or signs inside a store that could be visible from the street.

### Database Verification

In the absence of any official records, we used several verification methods to help determine if our online methods yielded accurate and up-to-date information. First, after we finished collecting data solely from the Internet sources, we called a random subset of dispensaries (approximately 10%) to verify information collected from the websites. As a second verification check, we compared dispensary information we collected from websites to the only known official available list of medical marijuana dispensaries in Los Angeles. As noted, currently most stores in Los Angeles County are unregulated and do not have business licenses. Yet in 2007, the city of Los Angeles passed an ICO that prohibited new medical marijuana dispensaries from opening but it allowed exemptions for stores that were already operating prior to the passing of the ICO and that were registered with the Los Angeles City Clerk (“Pre-ICO”). In 2013, ballot measure Proposition D allowed these 134 pre-ICO stores to remain open, provided they fell outside designated proximity to schools, churches, and certain neighborhoods. Later in 2013, the Los Angeles City Attorney’s office made the list of these dispensaries publicly available. As this represents the only official document of established open dispensaries, we used this list as the second verification to confirm that our online methods had located all 134 dispensaries, with the caveat that the list was created in 2013. Third, we were unable to determine the open/closed status of 100 dispensaries from online methods alone. Although the lack of online information to clearly indicate whether a store was open was a good indicator that the store was closed, as a third validation check post-data collection, we called these dispensaries to verify whether they had indeed closed. Lastly, we searched the five websites in August 2017, six months after initial data collection was completed, to determine how many of the confirmed open dispensaries were still open, had the same name, and had the same contact information (i.e., address, phone). This information was collected to demonstrate whether point-in-time snapshots of dispensaries are necessary given turnover, relocation, and closing of dispensaries. Only Internet methods were used for this recheck; that is, we used the five websites along with Google Maps to determine if the stores were open or closed using the same methods described for the initial data collection (e.g., viewing the latest update to the dispensary menu, observing “closed” as indicated on Google Maps or Yelp).

## RESULTS

Our methods identified 942 medical marijuana dispensaries in Los Angeles County. We retained 872 unique dispensaries after removal of duplicate and triplicate records. Most duplicate and triplicate dispensaries differed in name, but had the exact same address, across the website databases. Three dispensaries had the same name, but different addresses, across websites due to name changes that were reflected on the website with the most current information. Using website sources only, we determined that 470 dispensaries were open, 289 were closed, 13 were delivery only, and 100 were of unknown open/closed status. Characteristics of the unique dispensaries by open, closed, and unknown open/closed status are found in Table 1. Characteristics of the 470 unique open dispensaries identified through the online methods are further summarized below.

Among the 470 unique open dispensaries, most (47%) were within the city of Los Angeles (including South, East, and West), followed by the cities of Hollywood (6%, including East, North, and West Hollywood), Van Nuys (4%), Pasadena (3%), and Compton (3%). WeedMaps was most effective at locating open dispensaries, finding 95% of open dispensaries. Where’s Weed and Leafly both located 68% of open dispensaries, Yelp located 41%, and StickyGuide located 36%. Forty of the open dispensaries were located on one website source only (most often WeedMaps), 115 on two websites, 152 on three websites, 91 on four websites, and 72 on all five websites. WeedMaps also featured the least number of closed or unknown status dispensaries, whereas Where’s Weed was most likely to have closed or unknown status dispensaries listed (see Table 1).

A green cross was the most popular signage icon, with 43% of open dispensaries featuring this image, followed by signs indicating “Pre-ICO” (7%), “Prop-D Compliant” (7%), and picture(s) of a marijuana leaf (5%). Two dispensaries featured the word “cannabis” on a sign, one featured the outline of a green bong, and one sign contained the phrase “420” (i.e., as part of the store name). Other signs featured images and wording that did not clearly specify the site as a marijuana dispensary: two had pictures of leaves that were not in the shape of a marijuana leaf, four featured a non-green cross (e.g., yellow, red), four featured a palm tree, and five featured other pictures or wording that were not indicative of marijuana (i.e., a crown, the word “delivery,” three diagonal lines, stars, and a green clover). Forty-one percent featured no sign. We were unable to determine if there was a sign present for 5% of stores because the Google Maps image of the location was time stamped as prior to the dispensary’s open date (indicating lack of reliability), the image was unclear or illegible, the dispensary was located in a suite inside a larger building, or the store front was not visible from the street view available.

We were able to determine the open date for 59% of dispensaries (n= 275), which ranged from 2000 to 2016. We obtained month and year of opening for 128 dispensaries, year alone for 95, and a range of “2007 or prior” for the remaining 52. This latter category was determined due to a posting on the website(s) sources that indicated it had been featured on the site since 2007 or if the store featured a “Pre-ICO” sign.

Regarding names of the open stores, the majority (89%) had names that were not indicative that the store sold marijuana, 4% had names that clearly indicated the store sold marijuana, and 7% had names that probably indicated the store sold marijuana. The most commonly used terms in the dispensary names were “collective” (n=190); “green” (n=103); “care,” “caregiving,” or “caregiver(s)” (n=100), “wellness” (n=44), “herbal” (n=27); and “organic” (n=25). Twenty-four dispensary names featured the word “cap,” (e.g., “Buddy’s 25 Cap”) which indicates the maximum price a dispensary has set for one-eighth ounce of their “top shelf” (high-grade) product.

### Data Verification

We called 116 stores picked randomly in the full database to confirm information obtained from the online sources. We confirmed that 83 of the 83 closed dispensaries were closed (e.g., no answer at address during regular business hours, phone not in service, different business answered at location), eight of the eight delivery-only services were delivery only (i.e., no store front), and 24 of the 25 open dispensaries were open, with signage also confirmed. We were not able to confirm information for one open dispensary; after three attempts at calling there was no answer. For all closed dispensaries where the line rang with no answer after three attempts, we further confirmed the store was closed by using a Google Maps image search to ensure the store was not currently located at the specified address.

Using the list of 134 licensed dispensaries from the Los Angeles City Clerk in 2013, we found that our methods located 111 of these dispensaries (83%). Of these 111, 38 had the same name and address on the official list as we found through our methods, 54 had the same name but a different address listed between the website sources and the city list, and 19 were confirmed to have changed their name since the 2013 list. Twenty-two of these 111 dispensaries were confirmed as closed. Of the missing 23 dispensaries that were on the city list but not found via our methods, 15 were determined to have closed through a Google search; five were unable to be confirmed as closed or open through our website sources, Google search, or by calling phone numbers located from a Google search; one we could find no record of through a Google search of either the name or address; one was not found because it was not included in any of our website sources (but we found it through a Google search as open); and one was not found through our search, but this dispensary was listed on WeedMaps as open. For this latter one, however, the dispensary was added to WeedMaps during the month after we had completed the website’s extraction and conducted the verification check.

To verify the status of the 100 unknown open/closed status dispensaries, we called all 97 that had listed phone numbers and determined that these were closed if there was no answer after three attempts during business hours (n=41), the number was not in service (n=39), or there was a different business at the number (n=14). For three of these stores, someone answered and verified the store was open. For one of these three open stores, the clerk revealed that the store had a different name from the one that was listed on the marijuana websites.

**Figure 1.**
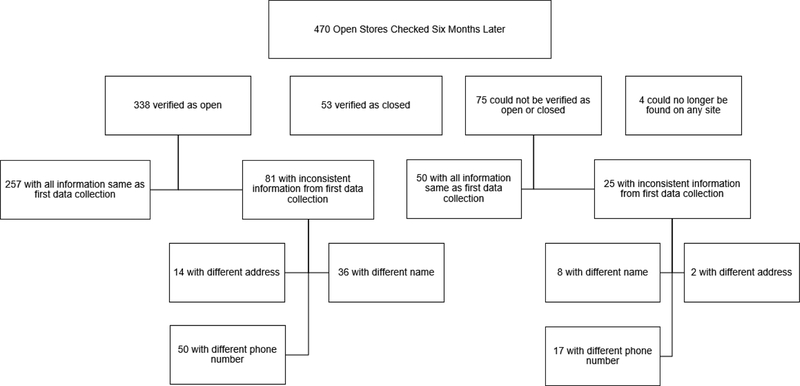
Use of Internet Methods to Recheck Information for the 470 Open Dispensaries

We also searched on the five websites for the open dispensaries six months after the initial data collection period ended. Of the 470 dispensaries that were confirmed as open during the initial data collection period, 53 (11%) were verified as being closed at the time of the recheck. An additional four (about 1%) dispensaries could not be located on any of the websites, nor through a Google search of the store’s name, address, or phone number. Seventy-five of the 470 dispensaries (16%) were located but could not be verified as open or closed using the five websites and Google Maps alone. The majority of the 470 dispensaries were verified as open (72%); however, 24% of these verified open dispensaries had information that was inconsistent from the first data collection period: 4% of the 338 open dispensaries had different addresses posted on the websites, 11% had different names, and 15% had different phone numbers. Several stores had more than one piece of information different from the first data collection period to the recheck six months later (e.g., both different address and different phone number). Figure 1 shows more details about these recheck findings.

## DISCUSSION

This is the first study to provide extensive detail on how to create a comprehensive and replicable database of medical marijuana dispensaries using data from online sources. Given that there is currently no official record of these marijuana outlets in Los Angeles County, we conducted a point-in-time data collection of dispensaries available on four medical marijuana dispensary search websites and one general business search website. We located 872 unique dispensaries, of which 470 were currently open. We verified our information by calling stores and reviewing our database against a 2013 official city list. Although our results and data verification outcomes speak to the thoroughness of our methods, it is clear that web-based observable methods alone may miss some dispensaries. For example, using the Internet alone we were not able to verify whether 100 stores were open or closed; thus, we needed to call these stores and inquire about their status. Interestingly, 97 of these 100 stores were verified as closed; thus, it may be reasonable to assume that if a store does not have an updated web presence, they may no longer be in business. In addition, there is much fluctuation in these dispensaries. One of our data verification procedures was to verify if contact information for the 470 open dispensaries changed six months after the initial round of data collection. We found that 11% had closed since our first data collection period and an additional 1% could no longer be located through any Internet search. Even though we found that 72% of the dispensaries that were open during the first data collection period were still able to be verified as open using the Internet methods alone, nearly a quarter of these had inconsistent information across the time periods, such as different names, addresses, or phone numbers. In all, only 55% (257 of 470) of the dispensaries at the recheck were verified as open and had all the same information from when we did the first collection. As these businesses fluctuate, the online dispensary search website sources also fluctuate. For example, two of the six website sources used by prior researchers to locate dispensaries in major cities in California have since shut down (Freisthler & Gruenewald, 2014) and one of our original website targets was taken down during the first month of data collection. This makes it clear that multiple website sources are needed in case a website goes offline or is not maintained.

In the absence of any official list from the county, these website sources appear to be the best source for updated and accurate information about dispensaries. Yet even with an official list, albeit four years old at the time of our data collection, dispensary location and name data did not always match what we found on the websites. For example, despite being able to determine that 83% of 134 dispensaries from an official Los Angeles city list had information on the websites, only 34% of these dispensaries had matching information (i.e., name, address) between the websites and the city list. Others had different names, addresses, or had been closed. Thus, extracting information from Internet sources may represent the most feasible and practical method for documenting the location of medical marijuana dispensaries in Los Angeles County at one point in time.

The WeedMaps website generated the most accurate and up-to-date information, locating 95% of open dispensaries in our database and featuring the fewest closed dispensaries on its site. This is likely because of the four marijuana websites we searched, WeedMaps is currently the largest and most used website. Although updated financial records are not available, the website was estimated to generate about $30 million in revenue in 2014 (Carreon, 2016). WeedMaps also has a free app that can be downloaded by customers, they are well advertised on billboards and social media (Bierut, Krauss, Sowles, & Cavazos-Rehg, 2017), and they are a major partner with the National Organization for the Reform of Marijuana Laws (NORML), which gives them a national presence. WeedMaps data have been used to answer important research questions, such as how dispensaries use WeedMaps to make claims about the health benefits of marijuana online, and whether underage youth are able to access information about marijuana when clicking from the website to dispensaries’ independent websites without needing to verify their age (Bierut et al., 2017). We found that using WeedMaps in conjunction with other website sources was helpful, as using Google Maps helped identify store front signage and determine open/closed status. However, it should be noted that advertising an unregulated/illegal business online comes with risks, and it is likely that some business owners opted not to include their information on WeedMaps, Yelp, or other websites. Business owners likely have to weigh the drawbacks of not appearing on the sites (e.g., losing out to more well-advertised competition, closing down because of no customers) against the risk of publicly advertising an unregulated business. As noted earlier, our methods may have missed dispensaries not appearing on any of these sites, but we cannot verify this in the absence of any official or accurate source for the location of these dispensaries.

We found that of the open stores, most did not have names that clearly discerned them as selling marijuana, and very few had signs besides green crosses that were clear indicators of marijuana. Of note, a limitation to using Google Maps to determine signage was that some signs were illegible and Google Maps only showed the street view of the store front, which could miss signage on the sides or back of businesses. Moreover, signage or store name alone are not the sole indicators of whether an individual is aware of a store’s presence in a neighborhood. For example, friends or relatives may alert someone to the presence of a store that is otherwise lacking a sign, customers may be loitering outside stores with paraphernalia or clothing referencing marijuana, and there may be a smell of the drug on the street outside the store from customers who have used the products before or upon exiting from the store. Though prior studies have not collected signage information, which no doubt has some effect on awareness of the dispensary’s existence in the neighborhood, studies that look at neighborhood density and proximity to these stores may need to also ask study participants if they are aware of dispensaries in their neighborhood regardless of whether they have or lack any clear signage or discernable store name.

Driving by locations is a method that may help to verify dispensary address and signage data. After first locating stores and addresses online, other researchers have driven to the posted addresses of medical marijuana dispensaries in Long Beach, California and Sacramento, California (Freisthler et al., 2016; Lipperman-Kreda et al., 2014). Researchers have also located “vape stores” that sold ENDS in two North Carolina counties by first locating them through online search engines and then driving on primary and secondary roads in the counties to confirm locations and look for stores selling ENDS that they may have missed (Lee, D’Angelo, Kuteh, & Martin, 2016). Such methods are possible in these smaller areas: Long Beach has a square mileage of 52 and Sacramento has a square mileage of 100. The two counties in North Carolina covered about 950 miles, but researchers only searched in populated (non-rural) areas. Such methods are not feasible in Los Angeles County as it covers over 4,750 square miles, with no largely unpopulated areas (e.g., average population per square mile is approximately 8,300 across Los Angeles County’s 272 neighborhood; see http://maps.latimes.com/neighborhoods/population/density/neighborhood/list/). In addition, because dispensaries fluctuate often and any database would need to be updated regularly, using online methods alone is more practical; although it likely misses some of the dispensaries that one may locate by driving every street in the county. Yet even these methods may miss dispensaries that choose not to have a street-side presence.

If driving to locations is not feasible, calling stores to verify information is another option for data collection. Researchers have also used online search engines such as Yelp, Google, and Yellow Pages supplemented by crowdsourcing to identify vape stores in the state of Florida that sold ENDS (Kim et al., 2016). Though the Internet search methods alone were mostly accurate in the study (e.g., Yelp identified 78% of stores selling ENDS) and represented a better system than using an outdated Florida state tobacco licensure list, the method was enhanced by using a crowdsourcing platform (Amazon Mechanical Turk [MTurk]) to call each store located from the online searches and confirm the store sold ENDS. MTurk workers took eight hours to call 1,459 stores and confirm information, and researchers replicated the eight-hour task two more times to verify accuracy across MTurk workers. Though costs of the tasks were not reported, this crowdsourcing methodology is no doubt more cost effective than driving to each location or having grant- or university-funded research assistants call each location and confirm information. Although we did not employ this methodology in the present study as we only called 10% of stores, it could be cost effective and practical for MTurk users to call the 872 dispensaries located in website searches and confirm open/closed status, verify addresses, and inquire about signage. Future studies may consider this approach.

To demonstrate feasibility of using the websites to document a point-in-time portrayal of the medical marijuana dispensaries in the area, we utilized a single coder for data extraction. This was meant to represent the typical extraction effort that may be completed on a small budget, perhaps by a research assistant or a graduate student collecting their own data for a thesis. Although the data verification procedures were conducted by two additional researchers, using an additional coder to extract data and documenting inter-rater reliability would have been a more rigorous (albeit time intensive) method of data verification at the extraction stage. It may also be possible to automate searches such that these websites are swept for current addresses, phone numbers, and store names, though signage information and current open status may be difficult to collect from automated methods. Researchers interested in using these methods should consider the effort needed to collect data from the other four websites beyond WeedMaps alone, as only 5% of the outlets we found on at least one website were not found on WeedMaps. If WeedMaps and Yelp were used alone, we would have located 464 of the 470 open outlets (99%). To locate the missing six outlets (1%), we would have needed to use Leafly and StickyGuide or Leafly and Where’s Weed. There were no outlets that were solely located on Where’s Weed or StickyGuide alone; thus, there was no unique benefit of using both of those sites after already using WeedMaps, Yelp, and Leafly.

More research is also needed to determine how comprehensive the websites can be in locating recreational marijuana outlets, as recreational sales are now legal in several states. Each of the marijuana websites we used can differentiate between medical and recreational outlets. Our methods focused on Los Angeles County alone to determine the feasibility of this approach in a large urban area covering over 4,750 square miles. Future studies can help determine if these methods are appropriate for locating marijuana outlets in other large cities or in more rural areas. The five websites used in this study cover many areas in the United States; for example, WeedMaps and Yelp cover all the states across the nation where medical (and now recreational) marijuana is available for legal purchases from dispensaries. StickyGuide currently allows searches for marijuana outlets in California (Bay Area, LA, Sacramento/Stockton), Colorado (Denver, Boulder, Colorado Springs), Maryland, Washington state (Seattle/Tacoma), and Washington, DC. Leafly is searchable for 27 states and Washington, DC, and Where’s Weed is searchable for 25 states and Washington, DC. As these websites widen their searchable areas, researchers across the United States can replicate our methods to help determine the feasibility and utility of this approach in their local areas.

### Conclusions

We acknowledge the prior work that has paved the way for our online methods of identifying medical marijuana dispensaries (Freisthler & Gruenewald, 2014; Freisthler et al., 2016; Hunt et al., 2014; Kepple & Freisthler, 2012; Kilmer et al., 2013; Nunberg et al., 2011; Pacula et al., 2002; Saloga et al., 2013; Thomas & Freisthler, 2016; Shi, 2016). Our work is innovative and moves the field forward in several ways. First, we have described our methods in detail, which makes replicability and generalizability possible. Methods could be used to develop databases of dispensaries in other large cities. Second, we included information about signage and name of store to describe if the dispensaries were clearly recognizable as medical marijuana outlets. This information is essential for researchers interested in determining mechanisms by which youth and adults may be influenced by proximity to dispensaries. Third, our methods indicated that it is important to maintain and update this database regularly given fluctuation of these businesses.

The methods presented here provide a structured guide to researchers to help them better identify dispensaries, which is crucial as availability of legal recreational marijuana continues to increase. Important caveats about the accuracy of data collected from online sources alone should be considered. Using online methods alone may be feasible and practical; however, use of other data sources and methods can improve the accuracy of these methods. These may include calling all located dispensaries to determine open status, driving by posted addresses to confirm address data, driving down populated streets to locate dispensaries not posted online and to verify signage information not available online, and comparing dispensaries located online to any available city, county, or state lists of licensed and unlicensed dispensaries. However, online methods may represent a best, practical effort for researchers seeking to locate dispensaries in a cost-effective way. Utilizing these methods, researchers can be better equipped to investigate how dispensary density changes over time, how these dispensaries associate with neighborhood factors such as crime and disorganization, and how proximity to and density of dispensaries may affect adolescent and adult marijuana use. Accurate information on dispensary names, locations, and signage can also provide important information on how marijuana availability may affect neighborhood quality, which can, in turn, inform policy decisions.

## Figures and Tables

**Table 1.** Characteristics of the 872 unique dispensaries located from the website methods

	Open (n = 470)	Closed (n = 289)	Unknown Open/Closed Status (n = 100)
*Website Source*	*Number of Dispensaries Located per Source*		
WeedMaps	446 (95%)	70 (24%)	17 (17%)
Where’s Weed	321 (68%)	204 (71%)	60 (60%)
Leafly	320 (68%)	86 (30%)	43 (43%)
Yelp	191 (41%)	124 (43%)	30 (30%)
StickyGuide	169 (36%)	187 (65%)	45 (45%)
***City Location of Dispensary***	*Number of Dispensaries Located per City*		
Los Angeles	221 (47%)	144 (50%)	59 (59%)
Hollywood	30 (6%)	15 (5%)	5 (5%)
Van Nuys	19 (4%)	24 (8%)	5 (5%)
Pasadena	14 (3%)	0 (0%)	1 (1%)
Compton	13 (3%)	6 (2%)	6 (6%)
Sylmar	10 (2%)	3 (1%)	0 (0%)
Wilmington	9 (2%)	7 (2%)	2 (2%)
Torrance	9 (2%)	4 (1%)	0 (0%)
Rosamond	8 (2%)	0 (0%)	0 (0%)
Sherman Oaks	7 (1%)	0 (0%)	2 (2%)
Studio City	7 (1%)	5 (2%)	0 (0%)
Canoga Park	7 (1%)	4 (1%)	2 (2%)
Sun Valley	7 (1%)	2 (1%)	0 (0%)
San Pedro	6 (1%)	9 (3%)	5 (5%)
Long Beach	1 (<1%)	10 (3%)	0 (0%)
Other	102 (22%)	56 (19%)	13 (13%)
***Signage***	*Number of Dispensaries with Type of Signage*		
Green cross	201 (43%)	7 (2%)	35 (35%)
Prop D Compliant	34 (7%)	1 (<1%)	1 (1%)
Pre-ICO	32 (7%)	1 (<1%)	1 (1%)
Picture(s) of marijuana leaf	26 (6%)	0 (0%)	2 (2%)
“Cannabis” or “420”	3 (1%)	0 (0%)	2 (2%)
Other	16 (3%)	1 (<1%)	1 (1%)
No signage	192 (41%)	10 (3%)	41 (41%)
Unable to be determined	26 (6%)	269 (93%)	21 (21%)
***Name of Store Indicates Sale of Marijuana***	*Number of Dispensaries with Name Type*		
Clearly indicative	18 (4%)	10 (3%)	2 (2%)
Probably indicative	32 (7%)	16 (6%)	7 (7%)
Not indicative	420 (89%)	263 (91%)	91 (91%)

*Note*. 13 dispensaries not included in the table above due to delivery only. Numbers within columns represent dispensary information prior to verification checks (e.g., the three unknown dispensaries that were determined to be open after a verification check are included in the unknown column).
